# Significant nocturnal wakefulness after sleep onset in metabolic dysfunction–associated steatotic liver disease

**DOI:** 10.3389/fnetp.2024.1458665

**Published:** 2024-12-04

**Authors:** Sofia Schaeffer, Andrijana Bogdanovic, Talitha Hildebrandt, Emilio Flint, Anne Geng, Sylvia Pecenko, Paul Lussier, Michael A. Strumberger, Martin Meyer, Jakob Weber, Markus H. Heim, Christian Cajochen, Christine Bernsmeier

**Affiliations:** ^1^ Department of Biomedicine and Department of Clinical Research, University of Basel, Basel, Switzerland; ^2^ University Centre for Gastrointestinal and Liver Diseases, Basel, Switzerland; ^3^ Centre for Chronobiology, Psychiatric Hospital of the University of Basel, Basel, Switzerland; ^4^ NovoLytiX GmbH, Witterswil, Switzerland

**Keywords:** MASLD, circadian misalignment, circadian rhythm, actigraphy, sleep hygiene education

## Abstract

Metabolic dysfunction–associated steatotic liver disease (MASLD) is a multisystemic disease with a multifactorial pathogenesis involving dietary, environmental, and genetic factors. Previous mouse models suggested that circadian misalignment may additionally influence its development as it influences metabolism in diverse organs including the liver. Further, data from sleep questionnaires proved sleep-wake disruption in patients with MASLD. We objectively assessed sleep-wake rhythms in patients with biopsy-proven MASLD (n = 35) and healthy controls (HC, n = 16) using actigraphy 24/7 for 4 weeks. With the aim to re-align sleep rhythms a single standardized sleep hygiene education session was performed after 2 weeks. Actigraphy data revealed that MASLD patients had more awakenings per night (MASLD vs. HC 8.5 vs. 5.5, *p* = 0.0036), longer wakefulness after sleep onset (MASLD vs. HC 45.4 min vs. 21.3 min, *p* = 0.0004), and decreased sleep efficiency (MASLD vs. HC 86.5% vs. 92.8%, *p* = 0.0008) compared with HC despite comparable sleep duration. Patients with MASLD self-reported shorter sleep duration (MASLD vs. HC 6 h vs. 6 h 45 min, *p* = 0.01) and prolonged sleep latency contributing to poorer sleep quality. Standardized sleep hygiene education did not produce significant changes in sleep parameters. Our findings indicate fragmented nocturnal sleep in patients with MASLD, characterized by increased wakefulness and reduced sleep efficiency, perceived subjectively as shortened sleep duration and delayed onset. A single sleep hygiene education session did not improve sleep parameters.

## 1 Introduction

Metabolic dysfunction-associated steatotic liver disease (MASLD) (Rinella et al., 2023) constitutes a growing global health burden due to its rising prevalence, affecting ∼25% of the world’s population (Younossi et al., 2018). MASLD is perceived as the hepatic manifestation of the multisystemic metabolic syndrome involving diverse human tissues, and involves a disease spectrum characterized by hepatic fat accumulation ranging from the more benign condition of the metabolic dysfunction-associated steatotic liver (MASL) to metabolic dysfunction-associated steatohepatitis (MASH) characterized by simultaneous inflammatory changes and hepatocytic damage such as ballooning that ultimately may progress to liver fibrosis, cirrhosis, and even hepatocellular carcinoma (Pouwels et al., 2022). The pathogenesis of MASLD is multifactorial with metabolic perturbations in diverse human compartments and regulatory systems (e.g., visceral obesity, arterial hypertension, dyslipidemia, insulin resistance), environmental and genetic factors, the microbiome, and comorbidities as the main determinants; however, a better understanding of the underlying mechanisms and interactions is desirable (Friedman et al., 2018). Specifically, a substantial body of experimental (Kettner et al., 2015; Fleet et al., 2016; Padilla et al., 2023; Buxton et al., 2012) and epidemiological evidence evolved implicating chronic circadian dysfunction in the pathogenesis of the metabolic syndrome, including hepatic steatosis development (Kim et al., 2013; Bernsmeier et al., 2015; Morris et al., 2016; Wijarnpreecha et al., 2016; Marjot et al., 2021).

The environmental light-dark cycle dictates most physiological processes in mammals. To adapt to this circadian rhythm, all mammals have developed a circadian pacemaker, the central circadian clock, which resides in the hypothalamus’s suprachiasmatic nucleus (SCN). It couples light input and the endogenous rhythm of peripheral tissues and works as a pacemaker (Welsh et al., 2010; Mukherji et al., 2019). Importantly, it coordinates physiological processes across multiple tissues (Saran et al., 2020). Increasing evidence suggests that synchrony between the SCN and peripheral clocks is mandatory for optimal health (Saran et al., 2020).

In murine models, deficiencies in circadian clock genes have been associated with hyperphagia, obesity, metabolic syndrome, and hepatic fat accumulation (Kettner et al., 2015; Fleet et al., 2016). Moreover, a disordered circadian rhythm has been shown to induce MASLD in offspring mice and even MASH-related carcinogenesis (Padilla et al., 2023; Mouralidarane et al., 2015). Additionally, changes in diet, sleeping patterns, or lighting conditions can result in the decoupling of central and peripheral clocks, inducing dysmetabolic states such as hepatic steatosis (Stenvers et al., 2019).

Similarly, there is an accumulating body of evidence from human experimental (Spiegel et al., 1999; Buxton et al., 2012), epidemiological (Kim et al., 2013; Bernsmeier et al., 2015; Morris et al., 2016; Wijarnpreecha et al., 2016; Marjot et al., 2021; Potter et al., 2016), and genetic (Sookoian et al., 2007) studies implicating an important role for the circadian clock and sleep cycle in the pathogenesis of the metabolic syndrome, specifically MASLD. Several observational studies based on self-reported sleep questionnaires have associated short sleep duration with MASLD (Hsieh et al., 2011; Kim et al., 2013; Bernsmeier et al., 2015; Imaizumi et al., 2015). Nocturnal light exposure, such as night shift work, has been shown to cause misalignment of circadian rhythms (Razavi et al., 2019; Choi et al., 2022; Cipolla-Neto et al., 2014) and is associated with features of the metabolic syndrome (Cipolla-Neto et al., 2014; Heo et al., 2018). Our group previously reported shortened sleep duration, delayed sleep onset, and poor sleep quality in patients with MASLD compared with healthy subjects using data obtained from sleep questionnaires. Besides, daytime sleepiness was positively linked with biochemical and histologic surrogates of disease severity in that study (Bernsmeier et al., 2015). A more objective assessment, such as actigraphy, recommended by the American Academy of Sleep Medicine to assess sleep or circadian rhythm disorders (Smith et al., 2018), exploring sleep patterns in patients with MASLD, is yet to be reported. Further, as a consequence of these data, it has been suggested that resynchronization of the circadian system and increased sleep duration, e.g., sleep hygiene counseling in overweight adults, could prevent or reverse the development of dysmetabolic states (Longo and Panda, 2016; Tasali et al., 2022).

In this study, we aimed to evaluate sleep patterns in patients with biopsy-proven MASLD compared to healthy controls (HC) and patients with cirrhosis of other origins using actigraphy as an objective sleep assessment tool, and sleep questionnaires.

## 2 Methods

### 2.1 Study subjects

Between October 2019 and November 2021, we recruited 46 adult patients with biopsy-proven MASLD or liver cirrhosis of other origins from the University Centre for Gastrointestinal and Liver Diseases, Basel, Switzerland. The latter served as a comparator for the MASLD group, especially in subjects with MASH-related cirrhosis. Healthy individuals (n = 16) of a similar age with no evidence of primary sleep disturbance, liver disease, or metabolic disease served as controls (Table 1). Based on the histological results obtained <2 years before inclusion, the following subgroups were defined and analyzed: MASL, MASH, MASH-related cirrhosis, and cirrhosis of other origins. The exclusion criteria were age <18 years, pregnancy or lactation, shift work, suspected or proven obstructive sleep apnea (OSA), other primary sleep or psychiatric disorders, hepatic encephalopathy, previous acute hepatic decompensation, previous bariatric surgery, alcohol consumption (>20 g of pure ethanol/day), and neoplasia. All current medical histories were listed. The HC were screened for liver pathology using medical history, abdominal ultrasound, and liver function tests. After study enrolment, all candidates were followed up for 4 weeks. This study was approved by the local ethics committee (EK224/02; approved 20th September 2019; amendment approved 20th April 2020). Written informed consent was obtained from all the participants.

**TABLE 1:** Demographic, clinical and biochemical baseline characteristics of participants. MASLD, Metabolic dysfunction-associated steatotic liver disease; MASL, metabolic dysfunction associated steatotic liver; MASH, metabolic dysfunction-associated steatohepatitis; BMI, body mass index; NA, not applicable/available; NAS score, Non-alcoholic fatty liver disease activity score; ASH, alcoholic steatohepatitis; HBV, hepatitis B virus; HCV, hepatitis C virus; HOMA index, Homeostasis Model Assessment; ALT, alanine aminotransferase; AST, aspartate aminotransferase; INR, International Normalized Ratio; LDL, low density lipoprotein; HDL, high density lipoprotein; GFR, glomerular filtration rate; CRP, C-reactive protein; DLMO, Dim Light Melatonin Onset. Data are presented as the median (IQR). *p < 0.05; **p < 0.01; ***p < 0.001; ****p < 0.0001 indicate comparison to healthy control group. Mann-Whitney U tests.

Variables *(median, IQR)*	Healthy controls (n = 16)	MASLD (n = 35)	MASL (n = 11)	MASH (n = 16)	MASH with cirrhosis (n = 8)	Cirrhosis of other origin (n = 11)
Age (years)	61 (55–63)	58 (39–63)	54 (37–63)	54 (36–64)	60 (54–69)	58 (49–64)
Sex (m: f)	8:8	23:12	6:5	13:3	4:4	7:4
Weight (kg)	73.8 (60.3–84.6)	89.2 (81.5–109.3)^***^	83.6 (72.8–105)^*^	96.4 (86–114.3)^***^	86 (72.2–123)	78.3 (73.8–93)
BMI (kg/m^2^)	23.5 (20.7–25.8)	31 (28–36)^****^	29.2 (27–34.4)^****^	31.7 (28.4–36)^****^	31.8 (28.7–37.2)^****^	27.6 (24.1–31.1)^**^
Waist circumference (cm)	83.5 (78–91)	109 (97–119)^****^	101.5 (93–110)^**^	115 (98–121)^****^	116 (93–121)^***^	98.5 (90–111)^**^
Known Diabetes [%] treated with insulin [%]	0 [0] NA	12 [34]^*^ 3 [8.6]	4 [37]^*^ 1 [9]	4 [25] 1 [6.25]	4 [50]^**^ 1 [12.5]	0 [0] NA
NAS Score [%] 0–2 3–4 5–8	NA	2 [6] 13 [37] 20 [57]	2 [18] 7 [64] 2 [18]	0 5 [31] 11 [69]	0 1 [12.5] 7 [87.5]	NA
Child Pugh Score Child A (5–6) Child B (7–9) Child C (10–15)	NA	NA	NA	NA	7 [87.5] 1 [12.5] 0	9 [82] 1 [9] 1 [9]
Aetiology of underlying cirrhosis [%]	NA	NA	NA	NA	MASH 8 [100]	ASH 8 [82] HBV 1 [9] HCV 1 [9] ASH + HCV 1 [9]
Systolic blood pressure (mmHg)	122 (114–129)	131 (122–141)	129 (113–142)	135 (128–142)^**^	122 (110–130)	138 (120–146)
Diastolic blood pressure (mmHg)	76 (68–79)	78 (68–87)	77 (65–96)	83 (75–86)	67 (56–75)^*^	80 (74–86)
Fasting glucose (mmol/L)	5.2 (4.9–5.4)	6.3 (5.2–7.2)^**^	5.8 (5.1–7.1)^*^	6.1 (5.4–7.1)^**^	7.1 (5–10.5)	5.7 (5.5–6.2)^**^
Fasting insulin (µU/mL)	7.1 (5–9.4)	21.2 (15–35.4)^****^	16.2 (11.6–28.2)^***^	26.6 (16.5–59.8)^****^	28.3 (16.6–31)^***^	20.2 (17.9–54.6)^****^
HbA1c (%)	5.5 (5.4–5.8)	5.7 (5.4–6.2)	5.7 (5.5–6.2)	5.6 (5.3–6.3)	6.4 (5.2–7)	4.7 (4.4–5.6)^*^
HOMA Index	1.58 (1.09–2.26)	6.58 (3.97–11.02)^****^	4.74 (2.53–8.37)^****^	7.32 (4.27–15.22)^****^	6.56 (4.91–10.88)^***^	5.85 (4.13–13.35)^****^
ALT (U/L)	24 (15–27)	53 (37–90)^****^	54 (44–90)^****^	58 (40–104)^****^	39 (28–66)^**^	40 (24–57)^**^
AST (U/L)	23 (20–28)	38 (28–51)^****^	38 (29–53)^****^	35 (27–51)^***^	40 (26–55)^**^	44 (31–83)^****^
Bilirubin (µmol/L)	7.1 (6.5–10.6)	8.6 (5.7–9.9)	7.1 (5.1–9.9)	9.1 (4.6–16.9)	9.5 (8.6–14.7)^*^	15.9 (11.8–22.8)^***^
INR	1 (0.9–1)	1 (0.9–1)	1 (0.9–1)	1 (0.9–1)	1.05 (1–1.1)	1.1 (1–1.2)^**^
Triglycerides (mmol/L)	0.83 (0.68–1.05)	1.69 (1.3–2.62)^***^	1.71 (1.35–2.99)^**^	2.07 (1.23–2.66)^**^	1.51 (1.07–2.22)^*^	1.04 (0.59–1.32)
Total cholesterol (mmol/L)	5.8 (4.6–6.0)	4.3 (3.5–4.9)^**^	5.0 (4.6–6.0)	4.2 (3.5–4.8)^**^	3.7 (3.1–4.3)^***^	4.1 (3.4–5.0)^**^
LDL cholesterol (mmol/L)	3.3 (2.5–3.6)	2.2 (1.4–3.0)^**^	3.0 (2.1–4.0)	2.1 (1.2–2.6)^*^	1.8 (1.3–2.3)^***^	2.4 (1.5–2.6)^**^
HDL cholesterol (mmol/L)	1.7 (1.4–2.0)	1.1 (1.0–1.4)^****^	1.2 (0.0.9–1.6)^**^	1.1 (1.0–1.2)^****^	1.2 (0.9–1.4)^**^	1.4 (1.2–1.5)^*^
Creatinine (µmol/L)	77 (70–90)	73 (61–84)	79 (64–92)	75 (60–86)	65 (59–78)	66 (60–68)^*^
GFR (ml/min/1.73m2)	82 (74–92)	96 (83–110)^*^	87 (67–108)	100 (88–117)^**^	92 (80–108)	96 (84–101)^**^
Urea (mmol/L)	5.2 (4.1–6.1)	5.1 (4.5–5.9)	4.6 (4.4–6.8)	5.2 (4.5–5.5)	5.1 (4.1–6.3)	4.7 (4.0–6.0)
Uric acid (µmol/L)	325 (208–363)	327 (267–368)	331 (259–360)	330 (298–372)	291 (250–417)	345 (313–393)
Leukocyte count (10^9^/L)	5.6 (5.2–6.1)	6.7 (5.4–8.7)^*^	6.1 (5.6–7.95)	7.1 (5.4–9.4)^*^	6.6 (4.99–8.6)	5.7 (3.8–7.2)
CRP (mg/L)	0.7 (0.4–0.9)	2.1 (1.0–4.6)^****^	2.1 (0.6–6.7)^**^	2.0 (0.9–5.9)^***^	2.5 (1.5–3.7)^***^	1.8 (0.3–4.5)
Ferritin (µg/L)	144 (98–266)	156 (79–267)	87 (64–271)	208 (140–409)	106 (46–220)	205 (111–555)
FibroScan (kPa)	4.4 (3.7–4.9)	10.2 (6.6–17)^****^	7.9 (4.5–11.3)^**^	10.6 (7.4–17.4)^****^	15.2 (9.1–149)^****^	20.9 (10.1–156)^****^
Cortisol basal (nmol/L)	419 (321–488)	346 (267–425)	409 (267–549)	346 (324–421)	254 (135–348)^**^	430 (211–511)
Melatonin at bed time (pg/mL)	15.05 (2.5–27.7)	10.0 (1.8–37.5)	8.22 (2.2–31.1)	21.5 (3.5–38.3)	3.7 (1.1–34.1)	6.63 (1.6–9.5)
DLMO (hours before bedtime)	−2.5 (−3.8 – −1.2)	−2.1 (−3 – −1.3)	−2.1 (−3.7 – −1.2)	−2.0 (−2.5 – −1.2)	−2.7 (−3.0 – −2.4)	−2.2 (−3.0 – −2.1)

### 2.2 Study visits

**FIGURE 1:**
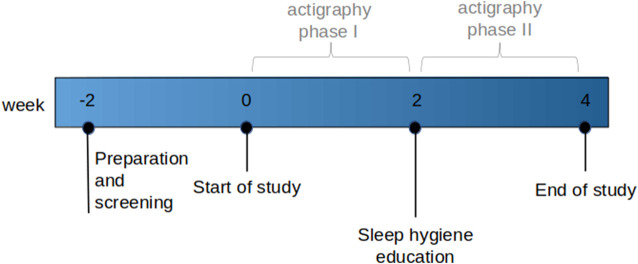
Course of the study.

All participants were scheduled for three study visits at baseline, week two, and week four, and the study was divided into actigraphy phase I (baseline to week two) and phase II (week two to week four; Figure 1). Week four was the end of the study period. In the case of inevitable appointment collisions, week two and week four appointments were shifted by a maximum of 1 week; in three cases, longer postponements were inevitable because of the lockdown during the COVID-19 pandemic. The baseline and end of the study visits were scheduled between 8 a.m. and 9 a.m. in a fasting state to assess sleep parameters and blood results simultaneously. At baseline and end of the study visits, vital signs, medication, personal history, blood count, INR, biochemistry, cortisol, and liver elastography (FibroScan^®^) were prospectively assessed. Additionally, three different sleep questionnaires (the Epworth Sleepiness Scale (ESS) (Johns, 1991), Karolinska Sleepiness Scale version B (KSS) (Baulk et al., 2001), and Pittsburgh Quality of Sleep Index (PSQI) (Buysse et al., 1989) were completed at baseline and the end of the study period. ESS with a cut-off >8 was used to screen for and exclude patients with OSA, the most common sleep disorder (Heinzer et al., 2015). KSS analyses the psycho-physical state experienced during the last 10 min. The PSQI assesses subjective sleep quality over 4 weeks, with a score >5 indicating poor sleep quality. Additionally, participants were asked to complete a daily sleep diary with information about excessive daily sleepiness (KSS), sleep latency, sleep disturbances, daytime sleeping, exercise, food, alcohol, caffeine, and nicotine consumption either in written or electronic form (SomnusApp^®^; Supplementary File S1).

A standardized sleep hygiene education (SHE) session was conducted between actigraphy phases I and II (week two). Participants were instructed to adhere to defined rules regarding healthy sleep habits, behaviors, and the adjustment of environmental factors with the intention of re-aligning their circadian rhythm (Supplementary File S2).

### 2.3 Melatonin saliva samples

To further elucidate the circadian rhythm in MASLD, all participants were instructed to take saliva samples for melatonin measurement using ELISA (Melatonin ELISA, MLTN-96, NovoLytiX GmbH, Switzerland) on Wednesdays in the second week of actigraphy phases I and II, respectively. Saliva collection started 4 h before bedtime using a plastic salivette (Salivette^®^, Sarstedt) and was repeated every hour until the assumed bedtime to obtain five samples. Participants were instructed to store the loaded salivettes at −18°C until the next study visit. The Dim Light Melatonin Onset (DLMO) was estimated using the “hockey-stick-method” to approximate the onset time of evening melatonin secretion (Danilenko et al., 2014). Under physiologic conditions, melatonin promotes sleep and is inversely secreted to light input with peak concentrations during darkness (Vasey et al., 2021).

### 2.4 Actigraphy and actigraphy analysis

Actigraphy using the ActTrust device (Condor Instruments, São Paolo, Brazil), a wrist-worn device for precisely measuring activity, light, and wrist temperature, was installed on the non-dominant wrist during the baseline visit. The candidates were instructed to wear the ActTrust device 24/7 for 4 weeks until the end of the study, except during showers, baths, or sports sessions.

The data recorded by ActTrust were analyzed using the ActStudio software (version 1.0.13). Following the recommendations of Condor Instruments, periods when the ActTrust was not worn and resting periods of less than 10 min were manually excluded from statistical analyses. The ActStudio software was used to calculate the following parameters: bedtime, get-up time, time in bed, total sleep time, sleep onset latency, sleep efficiency (%, total sleep time divided by the time in bed), number of awakenings, wakefulness after sleep onset (WASO, total time within a sleep period not in a sleep state), number, total time, and percentage of main and secondary sleep episodes. In addition, non-parametric circadian rhythm variables were calculated. These comprise inter-daily variability (IV) to characterize rhythm fragmentation, inter-daily stability (IS) showing synchronization to the 24 h light/dark phase, M10 defined as the most active 10-h period, nocturnal activity (L5) defined as the average activity during the least active 5 h, and relative amplitude (RA) representing the difference between M10 and L5. Actograms, graphical representations of an individual’s activity, and rest phases over a day were constructed using these results.

### 2.5 Statistical analysis

Actigraphy data were analyzed and graphed using ActStudio software (version 1.0.13). Prism 9.3.0 (GraphPad, La Jolla, CA, United States) was used for other data analysis and graphing. The Mann-Whitney or Wilcoxon tests were applied for data that did not follow a normal distribution. Correlations were analyzed using Spearman’s coefficients. *p*-value < 0.05 was considered statistically significant. Results are presented as median (interquartile range) unless otherwise specified. Box plots show the median with 10–90 percentiles.

## 3 Results

### 3.1 Patient characteristics

In total, n = 62 individuals were included. Details of baseline demographic, clinical, and biochemical characteristics are presented in Table 1. All patients with MASLD were obese (median BMI MASLD vs. HC 31 kg/m^2^ vs. 23.5 kg/m^2^, *p* < 0.0001; median waist circumference MASLD vs. HC, 109 cm vs. 83.5 cm, *p* < 0.0001), and 80% had manifestations of the metabolic syndrome. Fasting glucose and insulin levels and the HOMA index were significantly higher in patients with MASLD than in HC (Table 1). Moreover, patients with MASLD had significantly higher serum triglyceride levels than HC and patients with cirrhosis of other origin (MASLD vs. HC: median 1.69 mmol/L vs. 0.83 mmol/L, *p* = 0.0002; MASLD vs. cirrhosis: median 1.69 mmol/L vs. 1.04 mmol/L, *p* = 0.007). Total cholesterol, LDL, and HDL levels were higher in HC than in patients with MASLD and cirrhosis and remained within the normal range (Table 1). However, patients with MASLD who were taking lipid-lowering drugs were not excluded from the analysis. Fibroscan^®^ values in patients with MASLD correlated positively with the histologic fibrosis score (r = 0.5252; *p* = 0.002; n = 32). A minority of participants (16.1%) took sleep medications at irregular intervals (ranging from <1x to 3x/week).

### 3.2 Actigraphy data

**FIGURE 2:**
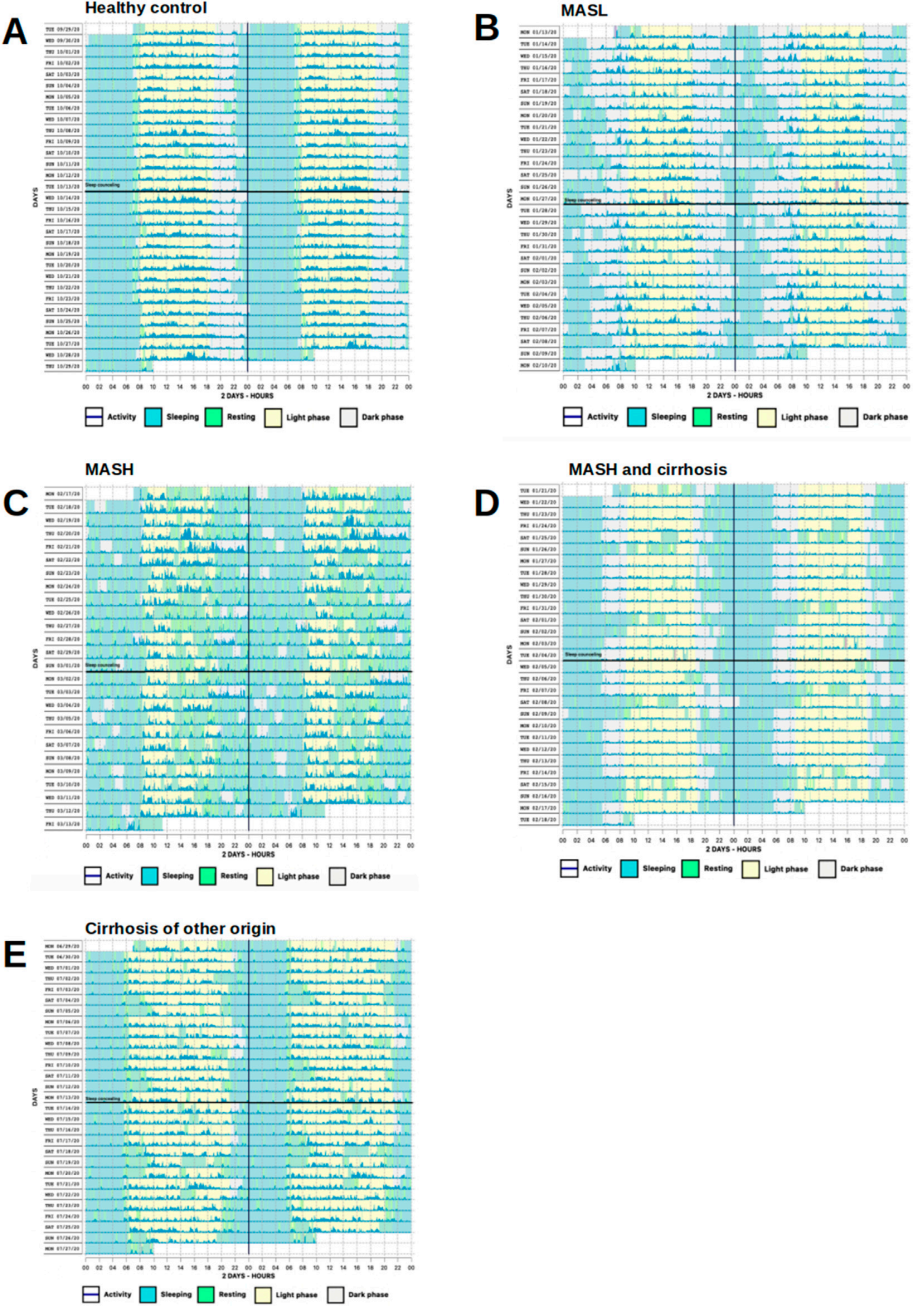
Representative actograms demonstrating phases of activity and rest over the course of a day. **(A)** Regular bed and wake-up times in a HC. **(B)** Irregular bed and wake-up times with multiple nocturnal awakenings in a MASL patient. **(C)** Completely disrupted day/night activity pattern and many diurnal sleep episodes in a MASH patient. **(D, E)** Irregular bed times, yet regular wake-up times and weekend compensation with daytime napping in a patient with MASH and cirrhosis and alcohol-related cirrhosis, respectively. Vertical black lines represent midnight, horizontal black lines SHE. One row represents 48 h. Awakenings during night are shown as green vertical lines within light blue and activity as dark blue lines. Sleep/wake patterns were detected by Condor instrument algorithm.

**FIGURE 3:**
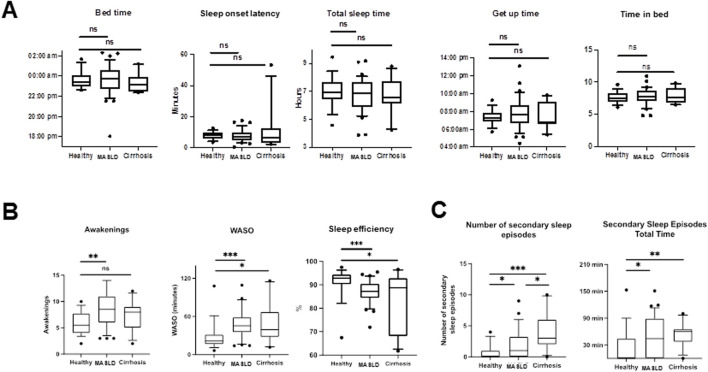
Comparison of sleep characteristics in different study groups determined by actigraphy recordings (actigraphy phase I). **(A)** Box plots showing sleep parameters during actigraphy phase I in HC, MASLD and cirrhosis patients. **(B)** Significantly more awakenings (MASLD vs. HC: median 8.5 vs. 5.5, *p* = 0.0036), longer WASO (MASLD vs. HC: median 45.4 min vs. 21.3 min, *p* = 0.0004) and reduced sleep efficiency (MASLD vs. HC: median 86.5% vs. 92.8%, *p* = 0.0008) were observed in MASLD patients in comparison with HC. **(C)** Number and total time of secondary sleep episodes is increased in patients with MASLD and cirrhosis of other origin compared with HC. ns not significant; *p < 0.05; **p < 0.01; ***p < 0.001. Comparison by Mann-Whitney U test.

N = 62 actograms were analyzed with representative examples shown in Figure 2. Contrary to previous findings based on subjective sleep questionnaires (Hsieh et al., 2011; Kim et al., 2013; Bernsmeier et al., 2015; Imaizumi et al., 2015), actigraphy data from patients with MASLD did not reveal significant differences in bedtime, sleep onset latency, sleep duration, get-up time, or time in bed (*p* > 0.05, Figures 3A, B). However, patients with MASLD had significantly more awakenings per night (MASLD vs. HC: median 8.5 vs. 5.5, *p* = 0.0036), and WASO was significantly prolonged, with a median time of 45.4 min vs. 21.3 min, *p* = 0.004, about twice as long compared with HC. Consequently, sleep efficiency was significantly lower in the MASLD group than in the HC group (Figure 3B). Interestingly, WASO was the highest among the MASL and MASH patients (Supplementary Figure S1A). Compared with patients with cirrhosis of other origins, the number of awakenings per night (MASLD vs. cirrhosis: median awakenings 9 vs. 8, *p* = 0.29) and WASO (MASLD vs. cirrhosis: median 45 min vs. 39 min, *p* = 0.52) were similar. In addition, there were no significant differences between patients with MASH cirrhosis and those with cirrhosis of other origins. The number of main sleep episodes (defined by the ActStudio program) was similar between patients with MASLD and HC, whereas some cirrhosis displayed more than one main nocturnal sleeping episode (cirrhosis vs. HC: median 2 vs. 1, *p* = 0.008; cirrhosis vs. MASLD: median 2 vs. 1, *p* = 0.01, Supplementary Figure S1A). All sleep parameters, nonparametric circadian rhythm analyses, and statistical analyses are presented in Table 2.

**TABLE 2:** Sleep parameters and non-parametric circadian rhythm analysis measured by ActTrust (Actigraphy phase I). MASLD, Metabolic dysfunction-associated steatotic liver disease; MASL, metabolic dysfunction-associated steatotic liver; MASH, metabolic dysfunction-associated steatohepatitis; WASO, wakefulness after sleep onset; M10, average activity during the most active 10 h period; L5, average activity during the least 5 h period; RA, difference between M10 activity and L5 activity ([M10-L5]/[M10 + L5]); IV, rhythm fragmentation; IS, synchronization to the 24 h light/dark phase. Data are presented as median (IQR) during actigraphy phase I. *p < 0.05; **p < 0.01; ***p < 0.001 indicate comparison with HC. #*p* < 0.05, compared with MASLD and and *p* < 0.05, refer to a comparison with MASH. Mann-Whitney U test.

Variables *(median, IQR)*	Healthy controls (HC, n = 16)	MASLD (n = 35)	MASL (n = 11)	MASH (n = 16)	MASH with cirrhosis (n = 8)	Cirrhosis of other origin (n = 11)
Bed time	23:22 (22:35–01:40)	23:42 (18:02–02:17)	23:27 (21:30–01:07)	23:51 (21:31–02:13)	00:36 (18:02–02:17)	23:06 (22:20–01:12)
Get up time	07:15 (05:42–09:16)	07:36 (04:24–13:07)	07:38 (04:24–09:21)	07:19 (05:56–09:12)	09:16 (05:07–13:07)	06:47 (05:23–09:50)
Time in bed (hours)	07:29 (06:05–09:37)	07:46 (04:48–10:54)	07:53 (04:48–10:54)	07:39 (04:49–09:13)	07:56 (06:13–10:08)	07:37 (06:29–09:49)
Total nocturnal sleep time	06:56 (04:34–09:28)	06:50 (03:51–09:10)	07:11 (03:51–09:05)	06:49 (03:54–07:53)	06:42 (05:23–09:10)	06:32 (04:13–08:47)
Sleep onset latency (minutes)	8 (3–12)	6 (0–17)	7 (0–10)	6 (2–10)	9 (4–17)	6 (2–53)
Sleep efficiency (%)	92.8 (67.4–97.5)	86.5 (71.8–95.4)^***^	85.3 (78.7–93.9)^**^	89.2 (71.8–95.4)^*^	87.3 (80–92.4)^**^	88.7 (61.7–96.5)^*^
WASO (minutes)	21 (6–108)	45 (14–146)^***^	57 (14–99)^**^	44 (14–146)^**^	44 (15–84)^*^	39 (11–167)^*^
Awakenings per night	5.5 (2–10)	8.5 (3–14)^**^	9 (3–13)^*^	8 (3–14)^*^	9 (4–14)^*^	8 (2–12)
Number of main sleep episodes per night	1 (1–2)	1 (1–2)	1 (1–2)	1 (1–2)	1 (1–2)	2 (1–2)^** # &^
Total number of secondary sleep episodes	0 (0–4)	1 (0–9)^*^	2 (0–6)	1 (0–9)^*^	1 (0–7)	3 (0–10)^*** #^
Secondary sleep episodes total time (minutes)	0 (0–02:33)	00:46 (0–04:25)^*^	00:49 (0–04:25)^*^	00:45 (0–01:57)^*^	00:21 (0–02:31)	01:00 (0–01:40)^**^
Total sleep time during 24 h period (including napping episodes)	07:05 (04:52–10:17)	06:58 (04:24–09:33)	07:29 (04:24–09:33)	06:50 (05:00–08:24)	06:48 (06:25–09:33)	07:03 (04:53–09:48)
Non–parametric circadian rhythm analysis						
M10	6506.6 (3253.3–6506.6)	5110.8 (2820.8–15567.1)	5328.1 (2820.8–7460.9)	5308.5 (3466.3–15567.1)	4572.6 (3035.0–5789.3)^* &^	4643.4 (1956.5–7996.3)
L5	49.5 (0–141.1)	48.6 (0–221.9)	39.2 (0–221.9)	64.6 (0–153.0)	30.5 (0–63.2)	49.5 (3.4–141.1)
RA	0.98 (0.92–1)	0.97 (0.89–1)	0.98 (0.89–1)	0.98 (0.94–1)	0.98 (0.97–1)	0.98 (0.92–0.99)
IV	0.7 (0.4–1.0)	0.7 (0.3–0.9)	0.6 (0.5–0.9)	0.6 (0.3–0.8)	0.6 (0.4–0.9)	0.6 (0.5–1.2)
IS	0.4 (0.2–0.6)	0.4 (0.2–0.5)	0.3 (0.2–0.5)	0.3 (0.2–0.5)	0.3 (0.2–0.4)	0.3 (0.1–0.6)

We further analyzed the diurnal sleep pattern during actigraphy phase I to investigate whether the reduced sleep efficiency observed in patients with MASLD was associated with previously described (Häusler et al., 2019) diurnal (secondary) sleep compensation. Patients with MASLD had significantly more absolute and longer diurnal sleeping episodes compared with HC (MASLD vs. HC: median 1 vs. 0, *p* = 0.02; median 46 min vs. 0 min, *p* = 0.03), similar diurnal sleeping episodes observed in patients with cirrhosis of other origin (cirrhosis vs. HC: median 3 vs. 0; *p* = 0.0005; median 66 min vs. 0 min, *p* = 0.004, Figure 3C).

The nonparametric circadian rhythm parameters were similar in the HC, MASLD, and cirrhosis groups (Table 2; Supplementary Figure S1B). Although HC self-reported being more active during the day than patients with MASLD or cirrhosis, only patients with MASH and cirrhosis displayed significantly lower M10 values (MASH with cirrhosis vs. HC: median 4418 vs. 6465, *p* = 0.03; MASH with cirrhosis vs. MASH: median 4418 vs. 5309, *p* = 0.014) (Table 2; Supplementary Figure S1B).

### 3.3 Questionnaires and sleep diary

**FIGURE 4:**
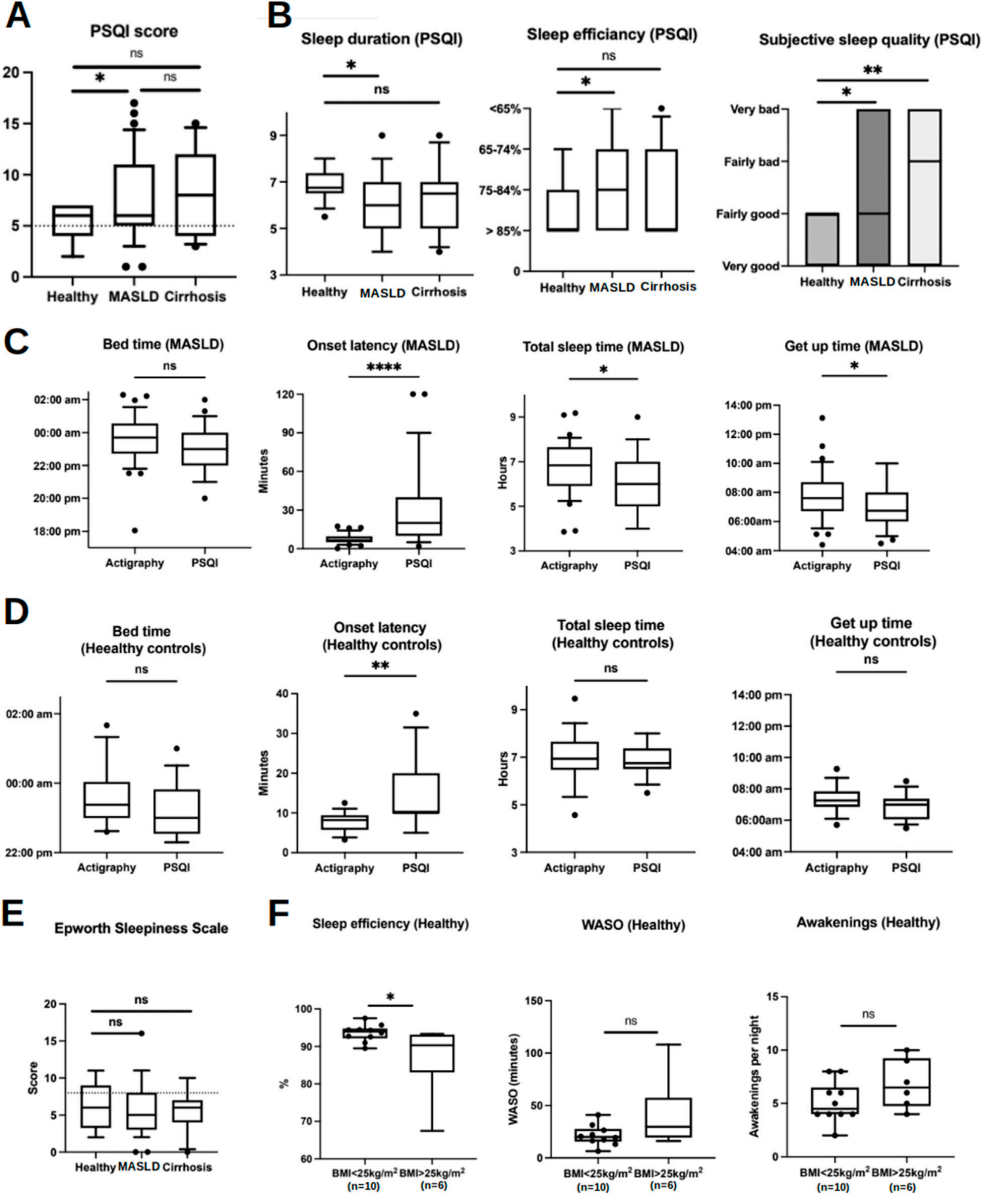
Sleep parameters acquired by sleep questionnaires in comparison with actigraphy data (at baseline and during actigraphy phase I). **(A)** Box plots show higher PSQI scores in MASLD patients compared with HC implicating worse subjective sleep quality over the 4-week period prior to study inclusion. Scores below the dotted line are considered as normal. **(B)** Box plots represent self-reported sleep parameters using the PSQI questionnaire. **(C, D)** Comparison of sleep parameters acquired by actigraphy and PSQI in MASLD patients and HC, respectively. **(E)** Results of ESS. The dotted line represents the cut-off of ESS 8 indicating presence of OSA. **(F)** Differences of sleep efficiency, WASO and awakenings in HC with or without overweight. Ns, not significant; *p < 0.05; **p < 0.01; ***p < 0.001. Comparisons by Mann-Whitney U tests.

The results of the sleep questionnaires at baseline are presented in Table 3. Patients with MASLD scored higher on the PSQI than the HC group, indicating reduced subjective sleep quality during the 4 weeks before study inclusion (Figure 4A). Looking at individual components of the PSQI score, patients with MASLD self-reported shorter sleep duration (MASLD vs. HC: median 6 h vs. 6 h 45 min, *p* = 0.01) and worse sleep efficiency (MASLD vs. HC: median 75%–84% vs. >85%, *p* = 0.01) compared to HC. Additionally, patients with MASLD and those with cirrhosis of other origins reported significantly worse subjective sleep quality than HC (MASLD vs. HC, *p* = 0.02; cirrhosis vs. HC, *p* = 0.003) (Figure 4B). According to the PSQI, the main reason for disrupted sleep was prolonged sleep latency (>30 min) (81.25% of HC, 71.5% of MASLD patients, 91% of patients with cirrhosis of other origins); however, no significantly prolonged (>30 min) sleep latency was recorded in any group by actigraphy. Other commonly reported causes of sleep disruption include going to the toilet experiencing excessive warmth, nightmares, and pain.

**TABLE 3:** Results of sleep questionnaires at baseline. MASLD: metabolic dysfunction-associated steatotic liver disease; MASL: metabolic dysfunction-associated steatotic liver; MASH: metabolic dysfunction-associated steatohepatitis; PSQI: Pittsburgh Sleep Quality Index; KSS: Karolinska Sleepiness Scale; ESS: Epworth Sleepiness Scale. Data are presented as the median (IQR) at baseline. *p < 0.05; **p < 0.01; ***p < 0.001 indicate comparison with HC. Comparison using the Mann-Whitney U test.

Variables *(median, IQR)*	Healthy controls (HC, n = 16)	MASLD (n = 35)	MASL (n = 11)	MASH (n = 16)	MASH with cirrhosis (n = 8)	Cirrhosis of other origin (n = 11)
PSQI score (scale from 0 to 21)	6 (4–7)	6 (5–11)^*^	8 (6–12)^*^	6 (4.25–8.75)	8 (3.75–12.75)	8 (4–12)
PSQI subjective sleep quality (scale from 0 to 3)	1 (1–1)	1 (1–2)^*^	2 (1–2)^**^	1 (1–1.75)	1 (0.25–2)	2 (1–2)^**^
PSQI sleep latency (minutes)	10 (8–20)	15 (5–30)	25 (10–40)	10 (6–22)	37.5 (11–60)^*^	15 (10–60)
PSQI sleep duration (hours: minutes)	6:45 (6:30–7:23)	6:00 (5:00–7:00)^*^	6:00 (5:00–6:00)^*^	6:15 (5:00–7:00)	5:30 (4:15–7:00)	6:30 (5:00–7:00)
PSQI bed time	23:00 (22:45–23:53)	23:00 (22:00–24:00)	22:00 (21:00–23:00)^*^	23:30 (22:26–24:00)	00:45 (22:26–02:23)	23:00 (22:30–24:00)
PSQI get up time	07:00 (06:04–07:23)	06:45 (06:00–08:00)	07:00 (06:00–07:00)	06:30 (05:49–08:00)	08:08 (06:08–10:00)	07:00 (05:40–08:00)
PSQI sleep efficiency (%)	93 (84.6–100)	80 (66–88)^**^	68 (64–80)^***^	84 (78.5–90)^*^	78.4 (60.6–90.1)^*^	85 (68–93)
PSQI use of sleep medication [%]	2 [12.5]	6 [17.1]	2 [18.2]	2 [12.5]	2 [25]	1 [9.1]
PSQI daytime dysfunction due to sleepiness (score from 0 to 3)	1 (0–1)	1 (0–1)	0 (0–1)	1 (0–1)	1 (0–2)	1 (0–1)
KSS alertness scale (from 1 to 9)	2 (1–3)	3 (1–4)	1 (1–3)	3 (1–4.75)	3.5 (1–7)	3 (1–3)
KSS sleep quality (scale from 1 to 5)	2 (2–2)	3 (2–3)^**^	3 (2–3)^**^	2 (2–3)	3.5 (3–4.75)^**^	2 (2–3)
KSS sleep latency (minutes)	10 (6–10)	15 (5–50)	20 (5–60)	13 (5–30)	40 (11–60)^*^	15 (5–120)
KSS awakenings	1 (0–2)	1 (0–2)	1 (0–2)	2 (1–2)	1 (0.25–1.75)	1 (0–2)
ESS Score	6 (3–9)	5 (3–8)	4 (2–7)	5.5 (4–9)	5.5 (0–11)	6 (4–8)
Average daily alcohol consumption (g/d)	5 (0–20)	0 (0–0)^***^	0 (0–0)^**^	0 (0–2)^*^	0 (0–0)^**^	0 (0–20)
Average daily coffee consumption (cups/d)	3.5 (3–5)	1.75 (0–2.625)^***^	1.5 (0–3.25)^*^	2 (0.25–3.75)^*^	1 (0–1.875)^***^	0.5 (0–2)^***^
Usual breakfast time	08:00 (7:30–10.30)	8:45 (7:50–10:15)	8:45 (7:53–9:00)	8:30 (7:38–9:00)	9:00 (7:40–12:00)	8:45 (7:30–9:53)
Usual lunch time	12:30 (12:00–12:45)	12:15 (12:00–12:53)	12:45 (12:00–13:08)	12:00 (12:00–12:30)	12:00 (11:53–12:08)^*^	12:30 (12:23–13:00)
Usual dinner time	19:00 (19:00–19:30)	19:00 (18:00–19:30)	19:00 (18:23–19:15)	19:00 (18:00–19:30)	19:30 (18:00–20:00)	19:00 (18:30–20:00)
Average physical activity per week (hours/week)	9 (6–10)	5 (0.5–26)	2.5 (0–10.75)^*^	9 (0.75–29)	5 (2–7)	8 (0–17.75)

Moreover, actigraphy revealed shorter sleep latency (actigraphy vs. PSQI: median 6:42 min vs. 20 min, *p* < 0.0001), longer sleep duration (actigraphy vs. PSQI: median 6 h 49 min vs. 6 h, *p* = 0.01), and later get-up time (actigraphy vs. PSQI: median 07:36 a.m. vs. 06:45 a.m., *p* = 0.03) than the self-reported data from the PSQI questionnaire in patients with MASLD (Figure 4C). HC also slightly overestimated their sleep latency (actigraphy vs. PSQI: median 08:25 min vs. 10 min, *p* = 0.002), whereas bedtime, total sleep time, and wake-up time were comparable between the objective and subjective methods (Figure 4D).

A fully completed daily sleep diary was returned by 84% of the participants. Similarly, sleep disturbances were reported by most participants, with environmental factors, pain, and nightmares being the main causes. 32% of patients with MASLD had disrupted sleep due to psychological stress, compared to only 6.25% of HC and 9% of patients with cirrhosis.

Disturbed sleep was commonly self-reported in all patients and HC when taken together; however, according to the PSQI, it led to a perceived lower sleep duration and quality only in patients with MASLD and cirrhosis.

Given that OSA has previously been described as a risk factor for the metabolic syndrome and MASLD (Bettini et al., 2022), we excluded patients with known OSA and screened them for possible undetected OSA. An ESS >8 as an indicator of undetected OSA (Heinzer et al., 2015) was reported in 24.2% of patients with MASLD; however, the overall ESS was similar in all study groups (Figure 4E). WASO, number of awakenings, and sleep efficiency did not differ among patients with MASLD with an ESS <8 and >8, and in patients who reported snoring (57.5% according to PSQI) compared to those who did not report snoring (42.5%) (Supplementary Figures S2A, B). Finally, WASO, awakening, and sleep efficiency were comparable in patients with MASLD when combining the two risk factors for OSA, ESS >8, and snoring, compared with those with ESS <8 and without snoring (Supplementary Figure S2C). In HC, 43% reported an ESS >8, and 56% reported snoring, suggesting a relatively high likelihood of undetected OSA. Similarly, ESS >8 and snoring did not confer higher WASO, more awakenings, or reduced sleep efficiency in HC (Supplementary Figure S2D). The level of fatigue, as assessed using the KSS, did not differ among the study groups (Supplementary Figure S2E).

Overweight (BMI 25–29.9 kg/m^2^), another risk factor for OSA, was prevalent in 37.5% (n = 6) of HC patients. The median BMI of the overweight HC was 25.95 kg/m^2^ compared with 21 kg/m^2^ in the other (n = 10). Interestingly, being overweight was associated with lower sleep efficiency, whereas WASO and the number of awakenings were unaffected (Figure 4F).

### 3.4 Association between sleep parameters and metabolic factors

We also investigated the association between metabolic factors and quantitative sleep parameters. Total sleep time was negatively correlated with BMI in patients with MASLD and HC (Supplementary Figure S3A). Total sleep time was also negatively associated with waist circumference in the HC (Supplementary Figure S3B), and in patients with MASLD, parameters of glucose metabolism correlated negatively with total sleep time and time spent in bed (fasting insulin, HOMA index, but not fasting glucose, Supplementary Figure S3C). In contrast, they were positively correlated with the number of awakenings (fasting glucose and HbA1c, Supplementary Figure S3D). In addition, there was a positive correlation between HbA1c levels and WASO, indicating decreased sleep efficiency (Supplementary Figure S3E).

### 3.5 Saliva melatonin measurement

**FIGURE 5:**
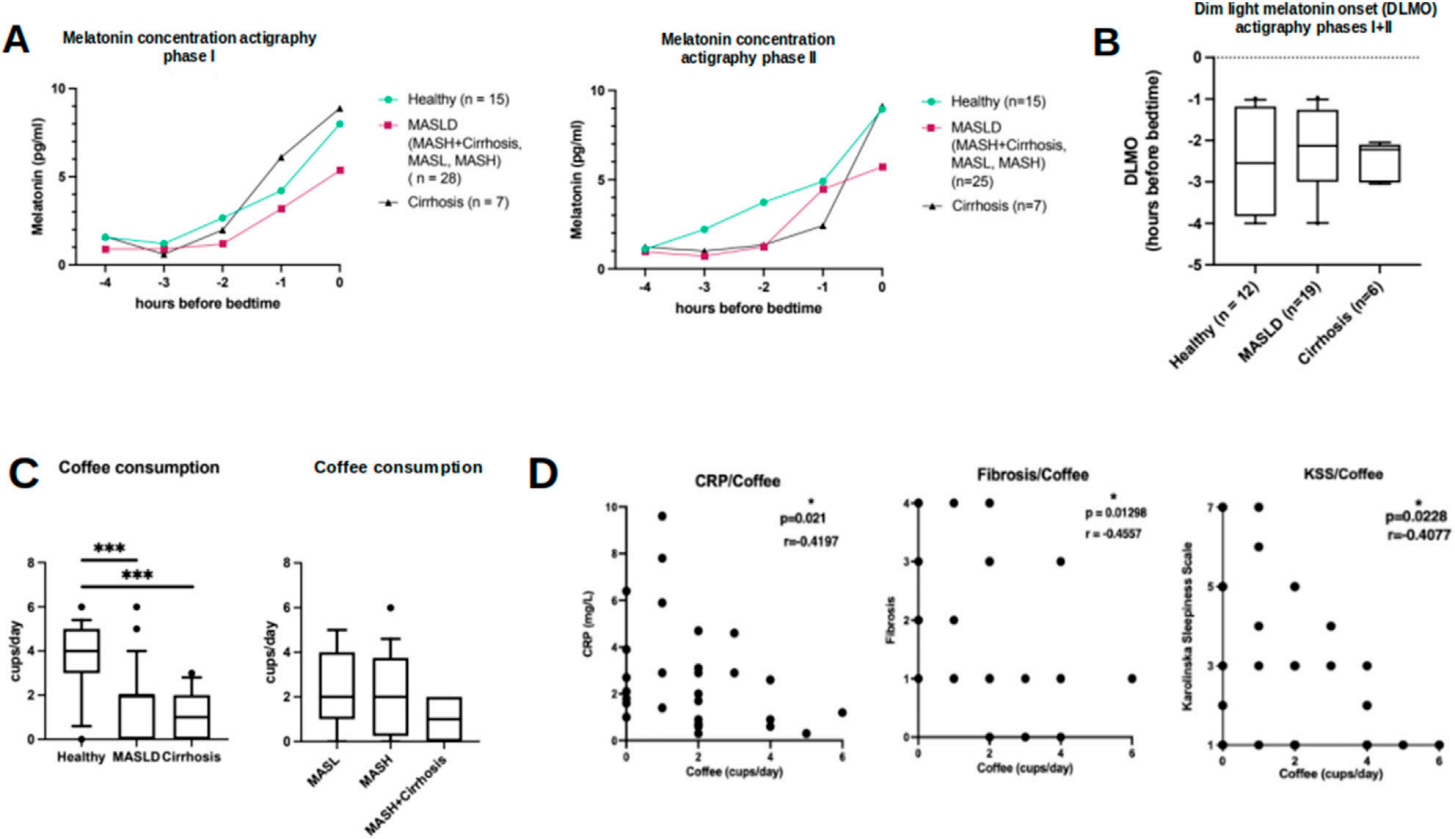
Results of saliva melatonin concentrations in relation to bed time and results of self-reported coffee consumption. **(A)** Saliva melatonin concentrations in HC (circle), patients with MASLD (square) and cirrhosis of other origin (triangle) in relation to hours before bedtime during actigraphy phase I (left) and II (right). **(B)** Similar onset of melatonin secretion (DLMO) during the whole study period in HC, patients with MASLD and cirrhosis of other origin. **(C)** Self-reported average coffee intake in HC, MASLD (subgroups on the right) and patients with cirrhosis of other origin. **(D)** Scatterplots show negative correlations between coffee consumption and CRP plasma levels, histologic fibrosis score and level of tiredness assessed by KSS questionnaire in MASLD patients. *p < 0.05; ***p < 0.0001. Mann-Whitney U test and Spearman correlation.

Salivary melatonin concentration results are shown in Figure 5A. Taken together, melatonin concentrations were comparable between patients with MASLD and controls. However, melatonin tended to increase less steadily in patients with MASLD, whose melatonin increase seemed less pronounced in both actigraphy phases I and II compared to HC and patients with cirrhosis of other origins. Subgroup analysis of the MASLD cohort revealed the lowest melatonin increase among patients with MASH scores and cirrhosis, whereas patients with MASH scores showed a significant yet irregular increase (Supplementary Figure S4A). Estimation of the approximate onset time of evening melatonin secretion (DLMO) showed no significant differences among the groups during the whole study period, despite a tendency of earlier start in melatonin rise among HC compared to patients with MASLD and cirrhosis (Figure 5B; Supplementary Figure S4B **)**.

### 3.6 Lifestyle habits

The reported daily consumption of coffee was significantly lower among patients with MASLD and among patients with cirrhosis of other origins than among HC (Figure 5C, MASLD vs. HC: 2 vs. 4 cups/day, *p* = 0.0004; MASLD vs. cirrhosis: 2 vs. 1 cup/day, *p* = 0.21; HC vs. cirrhosis 4 vs. 1 cup/day, *p* = 0.0001). Additionally, coffee consumption in patients with MASLD was negatively correlated with CRP levels (r = −0.41, *p* = 0.02, n = 30), histologic fibrosis score (r = −0.456, *p* = 0.013), and tiredness assessed by KSS (r = − 0.41, *p* = 0.023) (Figure 5D). However, these correlations were not observed in the HC group (data not shown).

Notably, 75% of HC self-reported drinking low amounts of alcoholic drinks (median 5 g of pure alcohol/day) compared to only 23.5% of MASLD (median 0 g/day) and 36.4% of cirrhosis patients (median 0 g/day) (*p* < 0.0001). However, these results need to be interpreted with caution, as patients with MASLD who drank more than 20 g of pure ethanol/day were excluded from the study. Moreover, patients with both MASLD and cirrhosis are actively urged not to consume alcohol, potentially impeding them from reporting the actual dose. Remarkably, only 35.5% of patients with MASLD reported practicing sports compared to 90.6% of HC (*p* < 0.0001). Concerning smoking (data not shown), meal times, and particularly dinner meal times, there were no significant differences between the patients and HC.

### 3.7 Effects of a singular sleep hygiene education session

**FIGURE 6:**
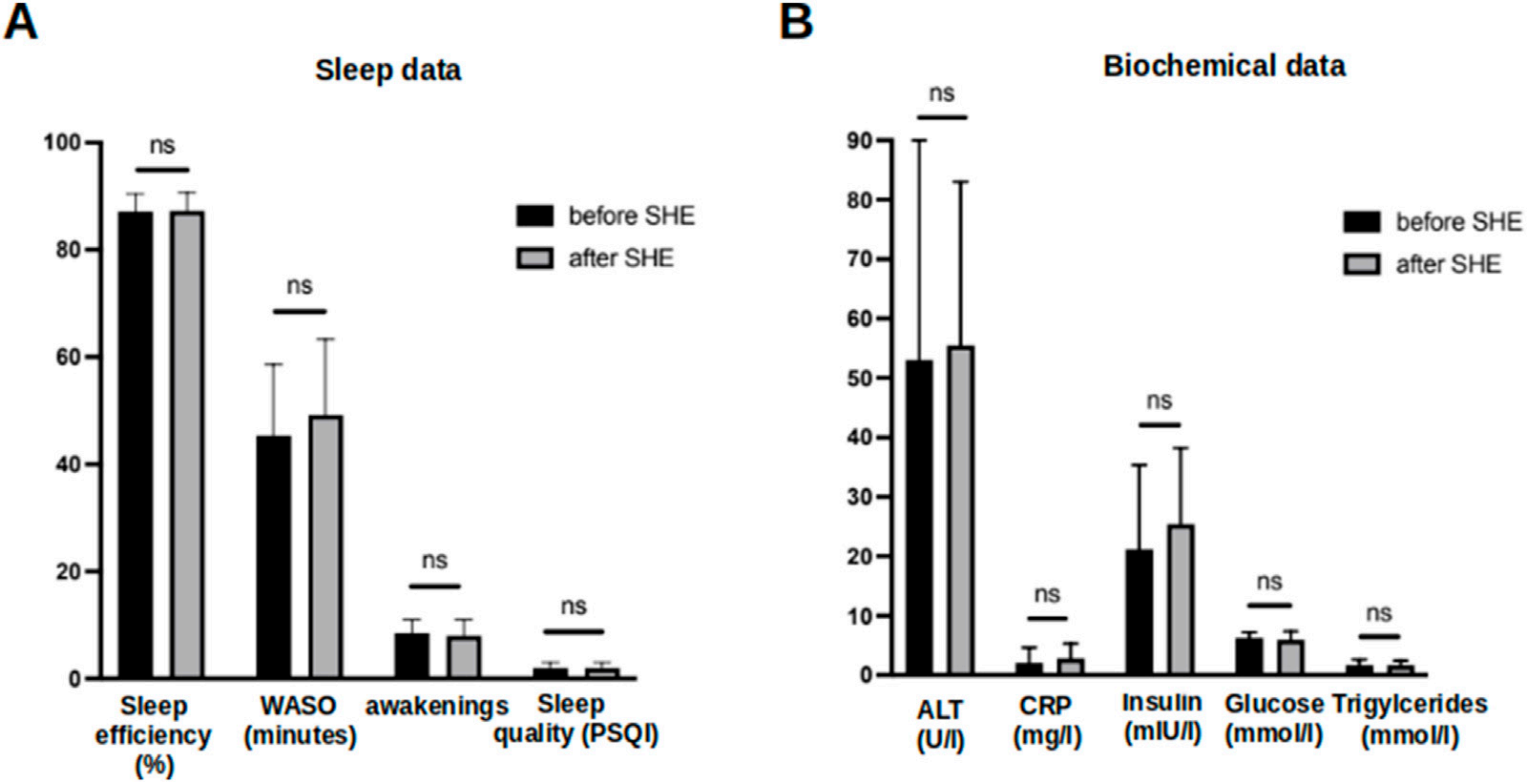
Comparison of objective and subjective sleep parameters and biochemical data before and after a singular session of SHE in MASLD patients. **(A)** Comparison of sleep efficiency, WASO, number of awakenings and subjective sleep quality assessed by PSQI questionnaire before and after a single SHE session in MASLD patients. **(B)** Comparison of biochemical data in MASLD patients at baseline and at the end of study. Ns–not significant. Comparison by Mann-Whitney U test.

Based on substantial evidence for the insomnia treatment (Chung et al., 2018), we performed a single SHE as a potential therapeutic intervention against sleep disruption in patients with MASLD. The participants learned about healthy sleep habits and were encouraged to follow recommendations for improving their sleep quality and quantity (Supplementary File S2). A single SHE session did not alter any of the objective actigraphy measures or subjective sleep parameters assessed using sleep questionnaires at the end of the study (Figure 6A). Note the similar sleep pattern findings in the actograms above and below the black horizontal line in Figure 2. Moreover, no relevant changes in the laboratory findings characterizing MASLD were observed at the end of the study, indicating no immediate changes in the metabolic status (Figure 6B).

## 4 Discussion

The present study evaluated quantitative and qualitative sleep characteristics in patients with biopsy-proven MASLD compared to a control group of healthy individuals and patients with liver cirrhosis of other origins using actigraphy. It revealed that patients with MASLD had longer nocturnal wakefulness, more fragmented sleep, and lower sleep efficiency. Although actigraphy assessed a similar time in bed (including episodes of wakefulness) and similar sleep onset in patients with MASLD and controls, patients with MASLD subjectively perceived and reported shorter sleep duration and delayed sleep onset, as reported in previous studies (Hsieh et al., 2011; Kim et al., 2013; Bernsmeier et al., 2015; Imaizumi et al., 2015).

Accumulating evidence supports an underestimated role of disturbances in the sleep-wake cycle relating to the pathogenesis of MASLD as hepatic manifestation of the multisystemic metabolic syndrome (Marjot et al., 2021). Actigraphy has been previously used to assess sleep in patients with liver cirrhosis (Córdoba et al., 1998; Montagnese et al., 2009). However, previous studies evaluating the association between circadian misalignment and MASLD applied uniquely subjective methods (i.e., sleep questionnaires (Buysse et al., 1989; Johns, 1991; Watson et al., 1988; Clark et al., 1988; Roenneberg et al., 2003). In contrast, here we present the – to our knowledge – first study applying actigraphy as a more objective method for analyzing sleep of patients with different stages of MASLD. Sleep analysis methods substantially influence the results and interpretations of clinical sleep studies. Generally, subjective information can be inaccurate and prone to reporter bias, whereas actigraphy is an established method for accurately estimating sleep time (Smith et al., 2018). Our results support the majority of findings of subjectively obtained sleep analyses. However, some differences were observed. Previous studies using subjective sleep assessment methods (Bernsmeier et al., 2015; Hsieh et al., 2011; Kim et al., 2013; Imaizumi et al., 2015) reported shorter total sleep duration in patients with MASLD than healthy individuals. Interestingly, although questionnaire data in our study confirmed this finding, actigraphy revealed similar sleep duration in patients with MASLD and healthy controls but was interrupted by a significant number and time of nocturnal awakenings. Several possible explanations exist for this subjective underestimation of sleep duration in patients with MASLD. First, despite comparable sleep duration, patients with MASLD experienced significantly longer nocturnal wakefulness and decreased sleep efficiency. These nocturnal awakenings mostly go unperceived. Subjectively, disrupted and inefficient sleep may be perceived as shorter sleep duration and suspended sleep onset or awakening. Second, compared to polysomnography, the gold standard for sleep assessment, actigraphy is less reliable in distinguishing rest from sleep, especially in subjects with low sleep efficiency (Alakuijala et al., 2021). Third, patients with MASLD reported significantly worse subjective sleep quality than HC (PSQI). Impaired subjective sleep quality may impair emotional health and, consequently, the general perception of sleep quantity and quality. This could also explain the overestimation of sleep latency and earlier wake-up times compared to actigraphy recordings among patients with MASLD. Furthermore, we observed more and longer diurnal sleeping episodes in patients with MASLD than in the HC. As previously described, this may result from increased nocturnal wakefulness and reduced sleep efficiency (Häusler et al., 2019).

Our data suggest a pathophysiological role for sleep fragmentation in developing MASLD. Previous evidence demonstrating a link between disturbances in the sleep cycle and diverse manifestations of the metabolic syndrome factors, such as glucose intolerance and obesity (Stenvers et al., 2019), was confirmed in the present study. With its pivotal role in fibrosis progression, insulin resistance may represent an important intersection between sleep disturbances and MASLD. Circadian disruption has been linked to increased levels of pro-inflammatory cytokines, such as interleukin-6, promoting hepatic fat accumulation and progression of MASLD (Marjot et al., 2021). Moreover, previous murine models demonstrated that genes involved in circadian rhythm control are also involved in energy metabolism, disrupting these genes and causing hepatic fat accumulation (Turek et al., 2005; Shimba et al., 2011; Chen et al., 2023). In humans, however, this reciprocal relationship remains unclear and needs further determination. Unfortunately, our study could not clarify the directionality of the translational relationship between disturbed sleep-wake patterns and MASLD.

Melatonin is an established marker of circadian function and an approved drug for modifying sleep patterns. Its role in energy metabolism and its antioxidant properties protecting against radicals have been well described (Cipolla-Neto et al., 2014). Further, animal and human studies have proven the protective effects of melatonin supplementation on several metabolic diseases, including liver injury (Yu et al., 2021). A previous study demonstrated higher fasting plasma melatonin concentrations in MASLD compared to healthy individuals (Sohrabi et al., 2021). Moreover, patients with liver cirrhosis reported higher daytime melatonin concentrations due to impaired hepatic melatonin metabolism and delayed increase in melatonin at night (Steindl et al., 1995). In contrast, salivary melatonin concentrations and DMLO in our study were similar in HC and patients with MASLD and cirrhosis of other origin, respectively. Further studies assessing melatonin in the saliva are needed to elucidate the course of melatonin in MASLD and to determine whether melatonin substitution may represent a therapeutic option for patients with MASLD.

Regarding lifestyle habits, our data corroborate the protective effects of coffee consumption on the development and progression of chronic liver disease, especially MASLD (Birerdinc et al., 2012; Bambha et al., 2014). The shift in meal intake towards late hours described in our previous study (Bernsmeier et al., 2015) was not confirmed here.

SHE is a common, non-pharmacological treatment for chronic sleep problems such as insomnia with controversial efficiency (Souilm et al., 2022; Irish et al., 2015). It involves adapting basic lifestyle habits that influence sleep, such as regular exercise, substance use, or sleep-wake times (Irish et al., 2015). To our knowledge, the effect of SHE in MASLD has yet to be studied. In our cohort, the lack of changes in objective or subjective sleep parameters after a single session of the SHE suggested resistance to change and improved sleep habits. This confirms previous data demonstrating the poor efficacy of SHE compared with cognitive-behavioral therapy for insomnia (Chung et al., 2018; Espie et al., 2019). Therefore, we suggest that future interventional studies targeting sleep efficiency in patients with MASLD should apply continuous sessions of SHE (instead of only one) or other treatment options.

Our study had several limitations. First, there was a significant difference in BMI between patients with MASLD and HC, representing a potential confounder that could explain the observed differences in sleep behavior. However, given that BMI >25 kg/m^2^ in HC was not related to nocturnal awakening, neither WASO argues against BMI as a cause of fragmented sleep in patients with MASLD. Unfortunately, due to considerable difficulties in recruiting an adequate number of overweight controls with no other manifestations of metabolic syndrome, specifically no liver steatosis, further studies are warranted to elucidate sleep behavior in obese individuals without MASLD. Second, OSA may be a confounding factor. OSA is a condition frequently observed in obese patients and has been described as a risk factor for MASLD (Bettini et al., 2022). Moreover, patients with OSA are known to have decreased sleep efficiency, more fragmented sleep, and shorter total sleep time, as measured by actigraphy (Otake et al., 2011). In our study, patients with OSA were excluded to the best of our knowledge and clinical practice, and the median ESS in patients with MASLD was normal and comparable to that in HC. However, our study did not perform polysomnography, the gold standard for diagnosing or excluding OSA, because of its complexity and high cost. Therefore, the individual patients included might still have undetected OSA. Nevertheless, the consistency of the results throughout the entire MASLD cohort, independent of ESS and externally reported snoring, argues against the presence of undetected OSA as an explanation for these results. Therefore, we hypothesized a more general association between sleep disruption and the development of MASLD. Further studies should consider polysomnography in their study protocols to better understand the impact of OSA on sleep disturbance in patients with MASLD. Third, the small number of included participants limited the interpretation and generalization of the data, especially in the MASLD subgroups. However, the strict exclusion criteria and the necessity of a long actigraphy period hampered the inclusion of a significantly larger cohort. Another consequence of the demanding recruitment process is that the age and sex of the cases and HC did not match perfectly. In addition, the methodology of melatonin sample collection was not directly observed, questioning the correct handling by patients and, consequently, the correctness of the data. Reporting, recall, and response biases may explain the discrepancies between actigraphy and self-reported data. Notably, the case-control study design limits the drawing of causal conclusions regarding the associations found.

In the present study, actigraphy was used as an objective measurement method to detect fragmented sleep patterns with significantly greater and longer nocturnal wakefulness in patients with MASLD. Sleep disruption led to a subjectively perceived sleep shortness. These findings support previous studies suggesting that disturbances in the complex regulatory network of the sleep-wake rhythm might play an important role in the pathogenesis of human MASLD. The attempt at a single behavioral therapy intervention did not lead to sustainable improvement in sleep efficiency for understandable reasons.

Further studies applying objective methods combined with subsequent interventions, such as repetitive sleep counseling sessions or melatonin supplementation, are warranted to evaluate the therapeutic potential of improving sleep efficiency in treating MASLD.

## Data Availability

The original contributions presented in the study are included in the article/Supplementary Material, further inquiries can be directed to the corresponding author.
