# Supplementary figures and images for: Significant nocturnal wakefulness after sleep onset in metabolic dysfunction–associated steatotic liver disease
Source: Front Netw Physiol. 2024 Dec 4;4:1458665. doi: 10.3389/fnetp.2024.1458665 (PMC11652136; doi:10.3389/fnetp.2024.1458665)

**A**

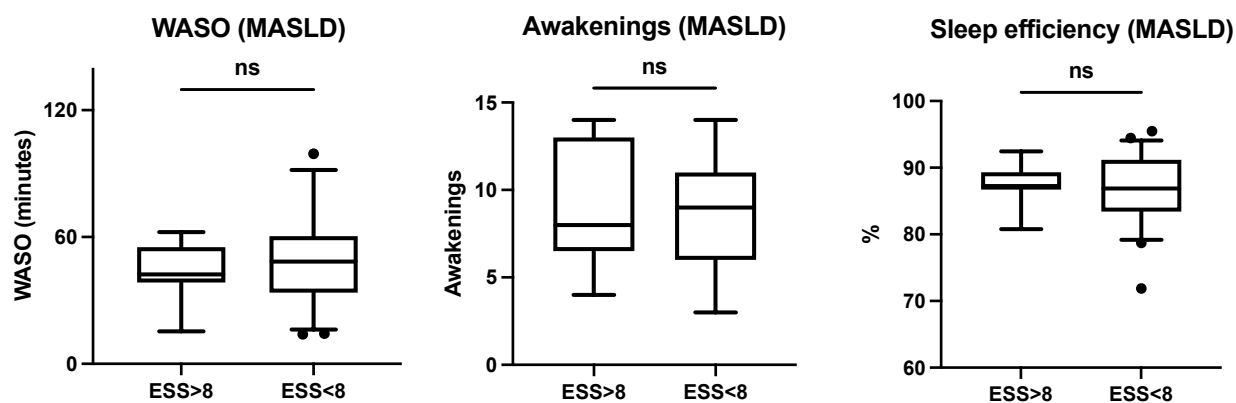

**B**

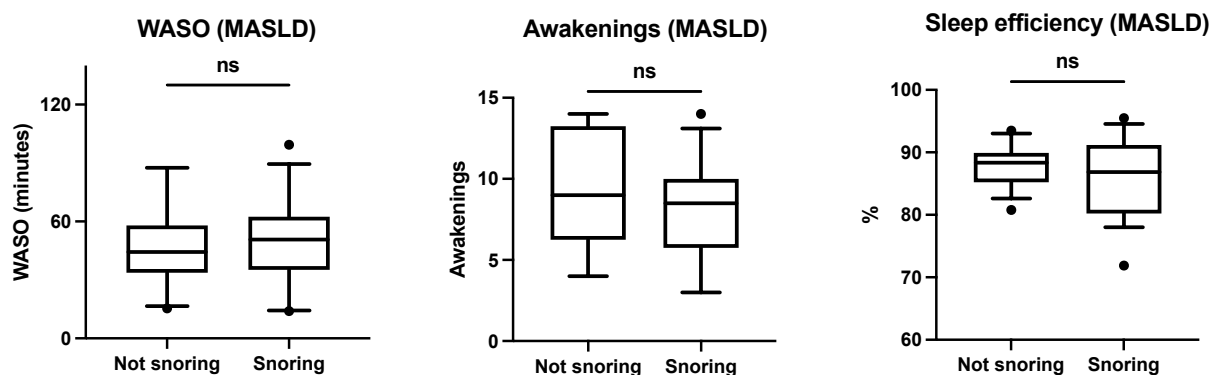

**C**

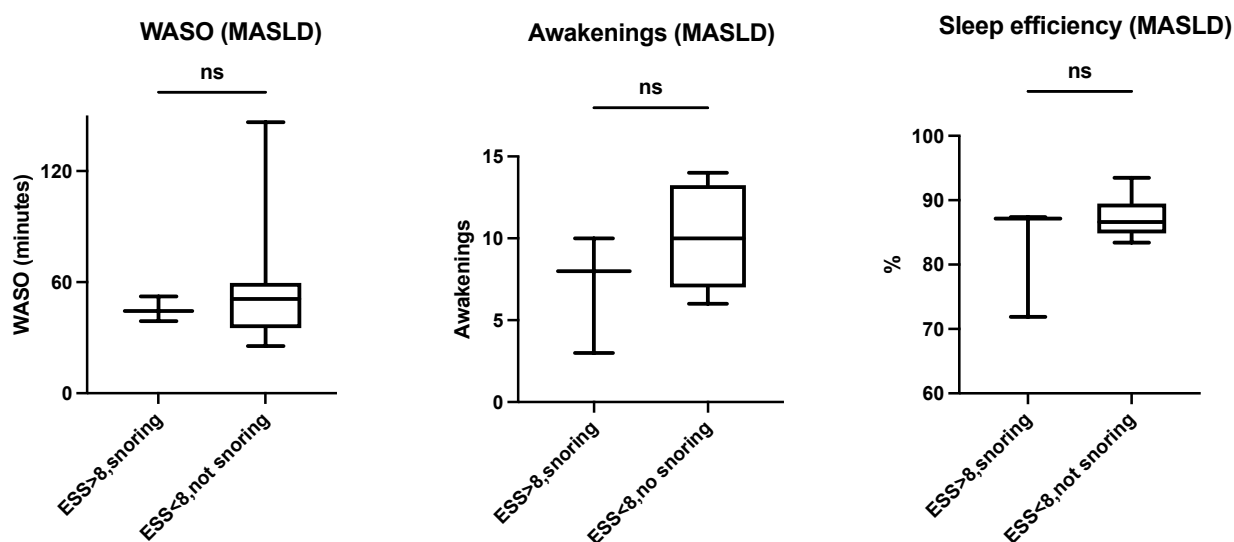

**D**

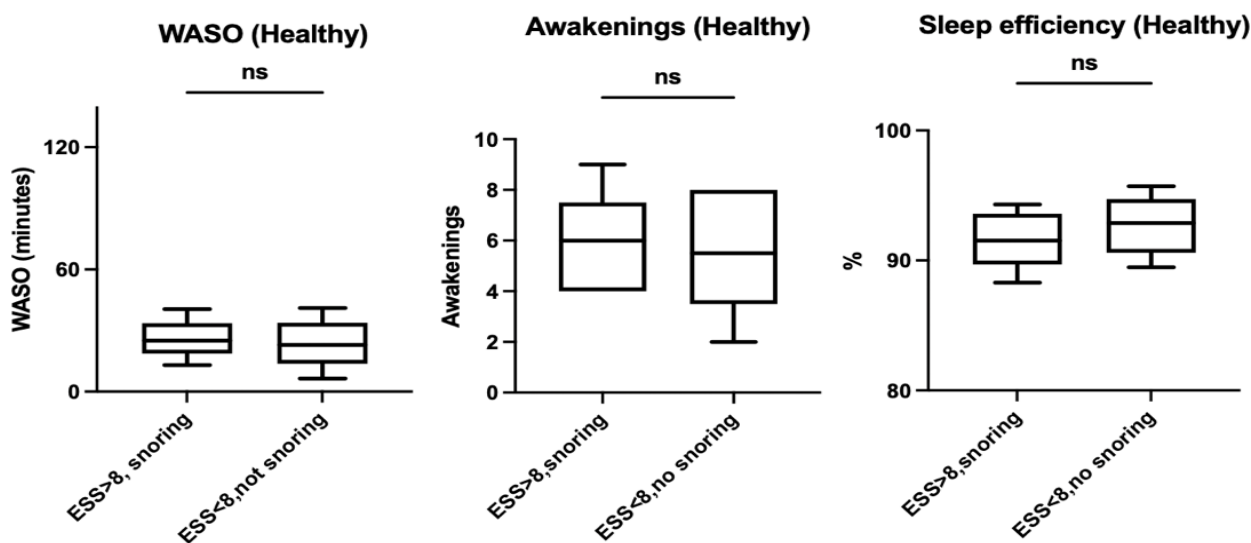

**E**

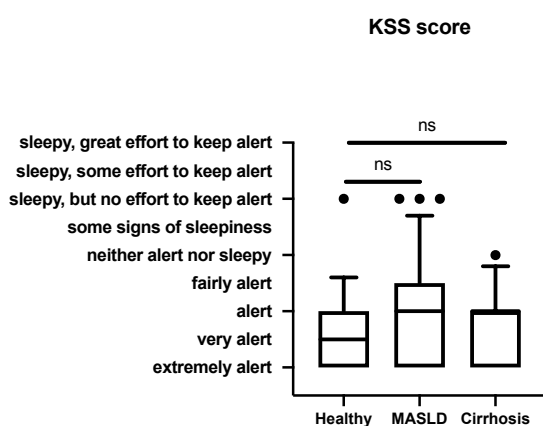

Supplement: Supplementary file 1 [file DataSheet2.PDF]

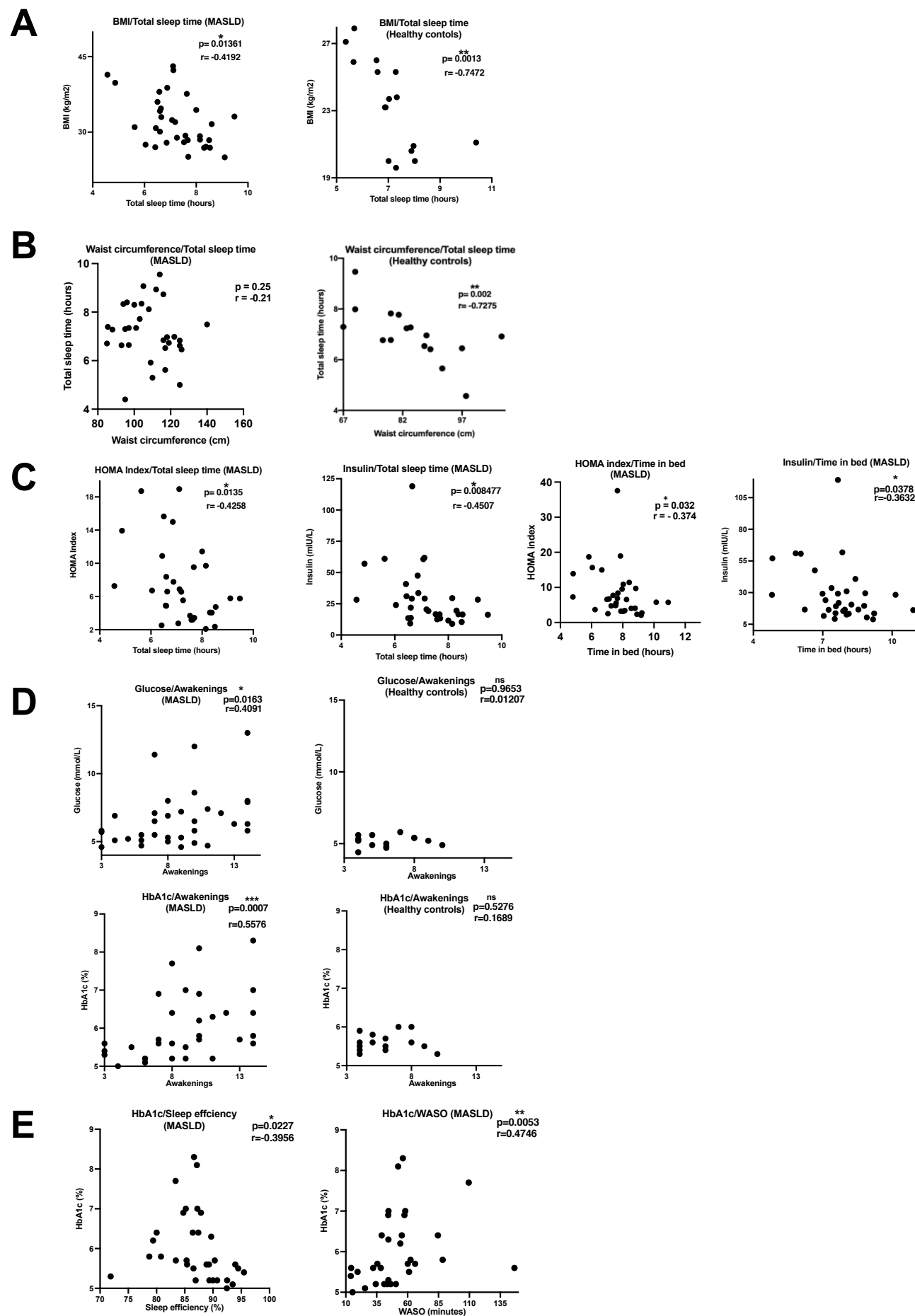

Supplement: Supplementary file 2 [file DataSheet4.PDF]

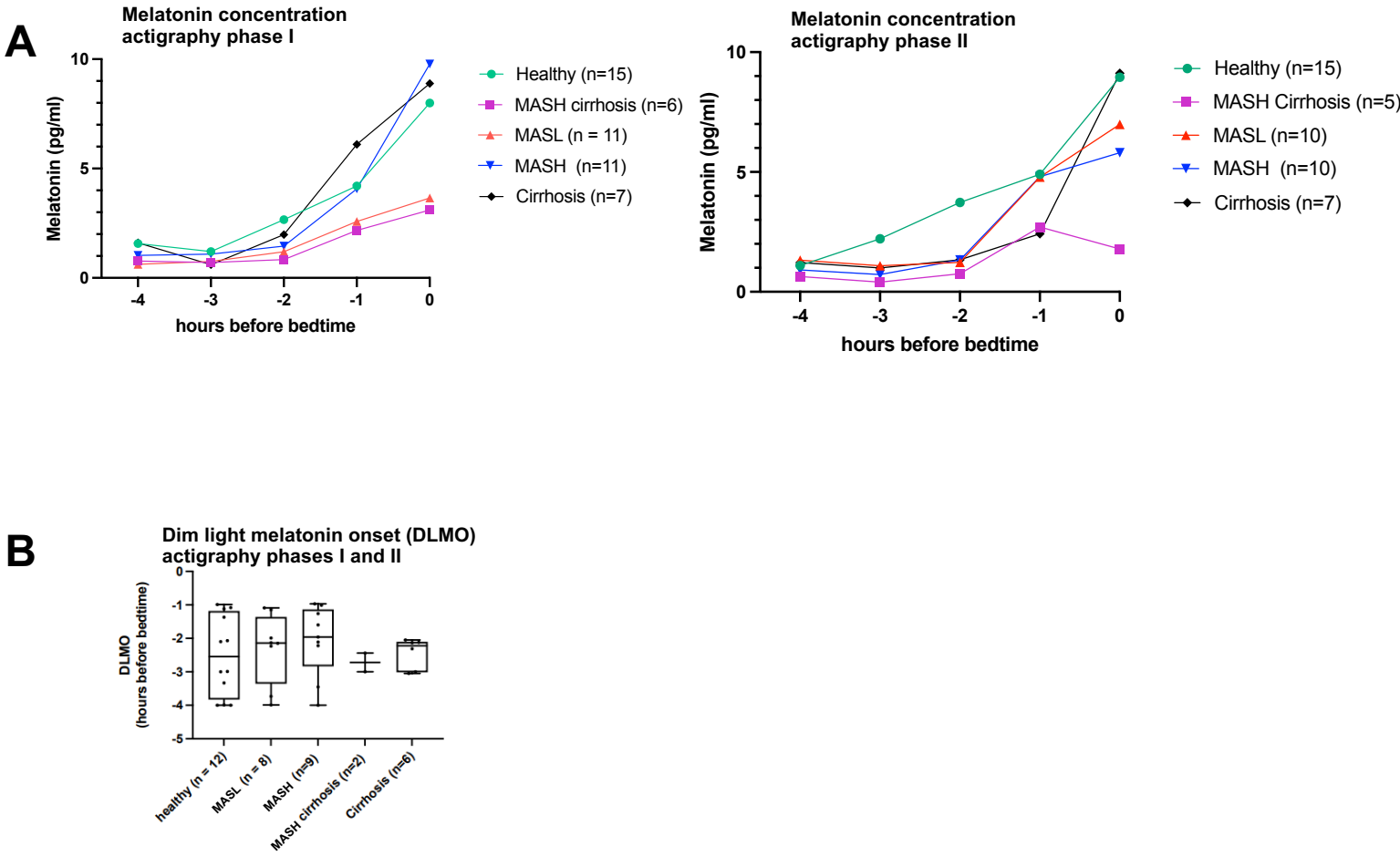

Supplement: Supplementary file 4 [file DataSheet3.PDF]

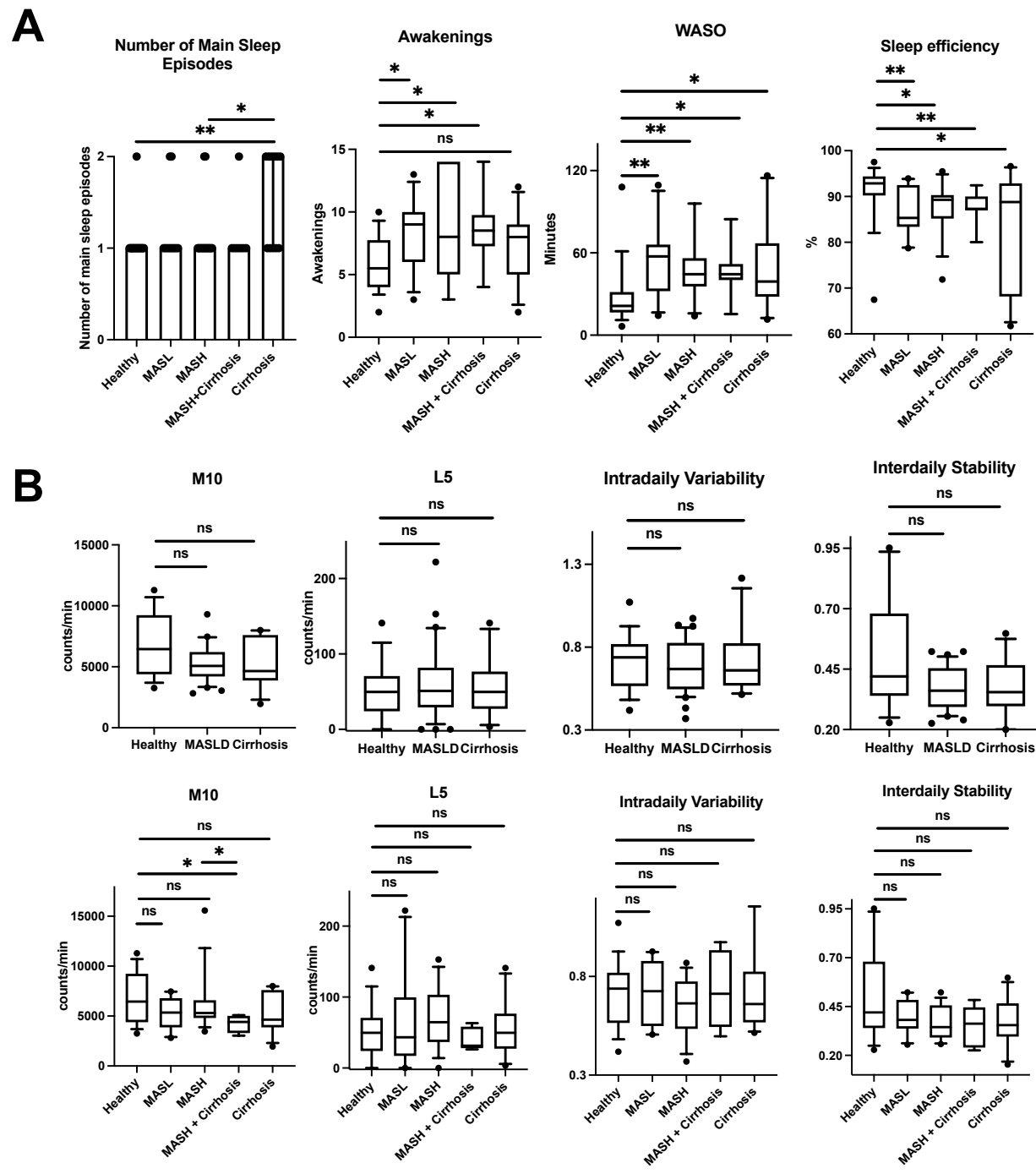

Supplement: Supplementary file 6 [file DataSheet1.PDF]
