# Supplementary material for: Significant nocturnal wakefulness after sleep onset in metabolic dysfunction–associated steatotic liver disease
Source: Front Netw Physiol. 2024 Dec 4;4:1458665. doi: 10.3389/fnetp.2024.1458665 (PMC11652136; doi:10.3389/fnetp.2024.1458665)
Supplement: Supplementary file 3 [file Table1.DOCX]

*************************

SLEEP – DIARY AND

INSTRUCTIONS FOR THE

CHRONOBIOLOGY

STUDY

Dear patient

Dear participant

ActTrust is a device that measures activity and sleep phases, ambient and skin temperature and light intensity. It allows us to gain deeper insights into everyday sleeping and waking behavior and also provide information about possible environmental disruptors that can have an unfavorable impact on healthy sleep. This device only measures presence/absence, but not type of activity.

While wearing this device, note:

1. Please wear the ActTrust device on your non-dominant hand (right-handed people wear ActTrust on the left, left-handed people wear ActTrust on the right) and if possible not under the sleeve because of the light sensor.
2. Please wear the ActTrust device continuously during day and night.
3. Do not wear the ActTrust device while showering or swimming as it is not waterproof. Don't forget to put it back on afterwards.
4. If you do intense sports (e.g. squash, tennis, handball), take off the ActTrust device and then put it back on.
5. If necessary, you can clean the ActTrust device with a damp cloth and mild soap.

If you have any questions, you can contact us at any time:

Andrijana Bogdanovic

Zentrum für Lehre und Forschung

Universitätsspital Basel

Hebelstrasse 20

CH - 4031 Basel

E-Mail: [andrijana.bogdanovic@unibas.ch](mailto:andrijana.bogdanovic@unibas.ch)

Name of the study participant:

S/N number of the ActTrust device handed out (Condor Instruments, Model: AT0503):____________

I hereby confirm that I have been informed both in writing (ActTrust information sheet) and verbally (by a member of the study team) about the appropriate use of the ActTrust device. I further certify that the ActTrust device will be returned to a member of the study team after use in the study project.

Place, date:_________________

Participant:__________________

**Instructions for the melatonin saliva samples**

Please read these instructions before starting saliva collection.

**When is a saliva sample taken?**

The collection takes place on Wednesday evening of the 2nd actigraphy week. A total of 5 saliva samples are taken:

- the first rehearsal 4 hours before going to bed

- then every additional hour (i.e. 3 hours, 2 hours and 1 hour before going to bed)

- the last (5th) rehearsal immediately before going to b

**Example:** If you go to bed around 10 p.m., take the first sample at 6 p.m., the second at 7 p.m., the third at 8 p.m., the fourth at 9 p.m., and the fifth at 10 p.m.

**How do you collect the saliva sample?**

1. Please write the time of sample collection on the tubes.

2. Please rinse your mouth thoroughly with water 15-20 minutes before taking the saliva sample.

3. Open the tubes with the cotton roll. There is an insert in the tube in which the cotton roll is located.

4. Place the cotton roll directly in your mouth without touching the cotton with your fingers.

5. Place the cotton roll between your teeth and cheek and move the cotton roll back and forth with your tongue for 3 minutes until the cotton roll is well soaked with saliva.

6. Return the cotton roll to the insert without touching it with your fingers.

7. Close the insert and return it to the tube. Please place the tube in the plastic bag and store it in the freezer until your next visit.

**Preparation for saliva collection:**

1. If possible, please do not eat anything for at least 30 minutes before taking the sample.
2. During the evening, please brush your teeth thoroughly without toothpaste after every meal and then rinse with plenty of water.
3. Please do not eat bananas and chocolate on collection day.
4. On collection day, please do not take aspirin and medications containing ibuprofen (Algifor, Brufen, Dysmenol, Dolocyl, Ecoprofen) as these medications can affect melatonin levels. If you are taking melatonin therapy, stop taking it for at least 3 days before collecting saliva.

- Please note on each tube the time and date the sample was taken and the sample number (1st sample, 2nd sample, 3….)
- Please bring the melatonin samples with you, preferably with cooling elements as the samples should be cold.

SLEEP-DIARY

Patient ID: __________________________________ Start date: ___________________ End date: ___________________

Please fill out this questionnaire between 8 a.m. and 9 a.m. every day.

|  | Date | | Date | | Date | | Date | | Date | | Date | Date |
| --- | --- | --- | --- | --- | --- | --- | --- | --- | --- | --- | --- | --- |
| 1. How would you rate your sleep last night? | **1 – Extremly good 2 – Good 3 – Neither good nor bad 4 – Bad 5 – Extremly bad** | | | | | | | | | | | |
|  | ①②③④⑤ | | ①②③④⑤ | | ①②③④⑤ | | ①②③④⑤ | | ①②③④⑤ | | ①②③④⑤ | ①②③④⑤ |
| 2. **Karolinska Sleepiness Scale (KSS)**  Please rate your tiredness in the last 10 minutes by checking the box in front of the corresponding number. Also use intermediate stages if applicable | ○ 1 (very awake)  ○ 2  ○ 3 (awake)  ○ 4  ○ 5 (neither awake nor tired)  ○ 6  ○ 7 (tired)  ○ 8  ○ 9 (very tired) | | ○ 1 (very awake)  ○ 2  ○ 3 (awake)  ○ 4  ○ 5 (neither awake nor tired)  ○ 6  ○ 7 (tired)  ○ 8  ○ 9 (very tired) | | ○ 1 (very awake)  ○ 2  ○ 3 (awake)  ○ 4  ○ 5 (neither awake nor tired)  ○ 6  ○ 7 (tired)  ○ 8  ○ 9 (very tired) | | ○ 1 (very awake)  ○ 2  ○ 3 (awake)  ○ 4  ○ 5 (neither awake nor tired)  ○ 6  ○ 7 (tired)  ○ 8  ○ 9 (very tired) | | ○ 1 (very awake)  ○ 2  ○ 3 (awake)  ○ 4  ○ 5 (neither awake nor tired)  ○ 6  ○ 7 (tired)  ○ 8  ○ 9 (very tired) | | ○ 1 (very awake)  ○ 2  ○ 3 (awake)  ○ 4  ○ 5 (neither awake nor tired)  ○ 6  ○ 7 (tired)  ○ 8  ○ 9 (very tired) | ○ 1 (very awake)  ○ 2  ○ 3 (awake)  ○ 4  ○ 5 (neither awake nor tired)  ○ 6  ○ 7 (tired)  ○ 8  ○ 9 (very tired) |
| 3. How long did it take you to fall asleep? | **1 - <10 min 2 – (10-20 min) 3 – (20 – 30 min) 4 – (30-60 min) 5 > 60 min** | | | | | | | | | | | |
|  | ①②③④⑤ | | ①②③④⑤ | | ①②③④⑤ | | ①②③④⑤ | | ①②③④⑤ | | ①②③④⑤ | ①②③④⑤ |
| 4. My sleep was disturbed by... | **1 - Nothing 2 – Noise 3 – circling thoughts 4 – waking up children 5 – work 6 - other** | | | | | | | | | | | |
|  | ①②③④⑤⑥ | | ①②③④⑤⑥ | | ①②③④⑤⑥ | | ①②③④⑤⑥ | | ①②③④⑤⑥ | | ①②③④⑤⑥ | ①②③④⑤⑥ |
| 5. Did you sleep during the day yesterday? | **1 – No 2 – Yes 3 – in the morning 4 – in the afternoon 5 - <30 min 6 – (30-60 min) 7 - >60 min** | | | | | | | | | | | |
|  | ①②③④⑤⑥⑦ | | ①②③④⑤⑥⑦ | | ①②③④⑤⑥⑦ | | ①②③④⑤⑥⑦ | | ①②③④⑤⑥⑦ | | ①②③④⑤⑥⑦ | ①②③④⑤⑥⑦ |
| 6. Did you do any exercise yesterday (at least 20 minutes)? | **1 – No 2 – Morning 3 – Afternoon 4 - Evening** | | | | | | | | | | | |
|  | ①②③④ | | ①②③④ | | ①②③④ | | ①②③④ | | ①②③④ | | ①②③④ | ①②③④ |
| 7. When did you have breakfast (first meal)? | **1 – Before 6 a.m. 2 – (6-7 a.m.) 3 – (7-8 a.m.) 4 – (8-9 a.m.) 5 – after 9 a.m.** | | | | | | | | | | | |
|  | ①②③④⑤ | ①②③④⑤ | | ①②③④⑤ | | ①②③④⑤ | | ①②③④⑤ | | ①②③④⑤ | | ①②③④⑤ |
| 8. When did you eat dinner (last meal)? | **1 – Before 6 p.m. 2 – (6-7 p.m.) 2 – (7-8 p.m.) 3 – (8-9 p.m.) 4 - after 9 p.m.** | | | | | | | | | | | |
|  | ①②③④ | | ①②③④ | | ①②③④ | | ①②③④ | | ①②③④ | | ①②③④ | ①②③④ |
| 9. Did you eat any “snacks” after dinner? | **1 – No 2 – yes, until 8 p.m. 3 – yes, until 10 p.m. 4 – yes, until midnight 5 - yes, after midnight** | | | | | | | | | | | |
|  | ①②③④⑤ | | ①②③④⑤ | | ①②③④⑤ | | ①②③④⑤ | | ①②③④⑤ | | ①②③④⑤ | ①②③④⑤ |
| 10. Did you drink caffeinated drinks (coffee, tea, cola, energy drinks) in the evening? | **1 – no 2 – until 8 p.m. 3 – (8-10 p.m.) 4 – (10 -12 p.m.)** | | | | | | | | | | | |
|  | ①②③④ | | ①②③④ | | ①②③④ | | ①②③④ | | ①②③④ | | ①②③④ | ①②③④ |
| 11. Did you drink alcoholic drinks in the evening? | **1 – no 2 – until 8 p.m. 3 – (8-10 p.m.) 4 – (10 -12 p.m.)** | | | | | | | | | | | |
|  | ①②③④ | | ①②③④ | | ①②③④ | | ①②③④ | | ①②③④ | | ①②③④ | ①②③④ |
| 12. Did you smoke in the evening? | **1 – no 2 – until 8 p.m. 3 – (8-10 p.m.) 4 – (10 -12 p.m.)** | | | | | | | | | | | |
|  | ①②③④ | | ①②③④ | | ①②③④ | | ①②③④ | | ①②③④ | | ①②③④ | ①②③④ |
| 13. Any special events in the last 24 hours? | ○ Yes ○ No  (Details) | | ○ Yes ○ No  (Details) | | ○ Yes ○ No  (Details) | | ○ Yes ○ No  (Details) | | ○ Yes ○ No  (Details) | | ○ Yes ○ No  (Details) | ○ Yes ○ No  (Details) |

**Installation of the „Somnus App”**

You can answer the daily questions on your sleep, activity and eating habits via Somnus App.

1. Find the Somnus App 2. Insert your username 3. Choose “I have 4. Insert your 5. Now you can answer

on Google Play. a Token” Token and your the questions!

new password.
