# Supplementary material for: Significant nocturnal wakefulness after sleep onset in metabolic dysfunction–associated steatotic liver disease
Source: Front Netw Physiol. 2024 Dec 4;4:1458665. doi: 10.3389/fnetp.2024.1458665 (PMC11652136; doi:10.3389/fnetp.2024.1458665)
Supplement: Supplementary file 5 [file Table2.DOCX]

**Sleep hygiene rules**

1. Maintain regular sleeping and waking times and, if possible, maintain the rhythm even on weekends.
2. Get as much daylight as possible every day, especially in the morning, at least 20 minutes!
3. Exercise in the day or evening (not too late) - this often works wonders.
4. Don’t go to bed “blue.” Today's smartphones, computer screens and LED home light installations emit a lot of blue light, which can have a disruptive effect on sleep.
5. Sleep in the dark if possible. TV sets do not belong in the bedroom because they also emit a lot of blue light (LED) and should not be used as a sleep ritual.
6. Words with friends can wait. End your chats 2 hours before your usual bedtime. Turn the device off or on airplane mode and leave it outside the bedroom.
7. Solve your problems before bed. If this is not possible, write them down. Then the mind can switch off better.
8. Don't smoke before going to bed.
9. Dinner should be light and eaten early in the evening.
10. Stop drinking too much in the evening and use alcohol, coffee and tea sparingly.
